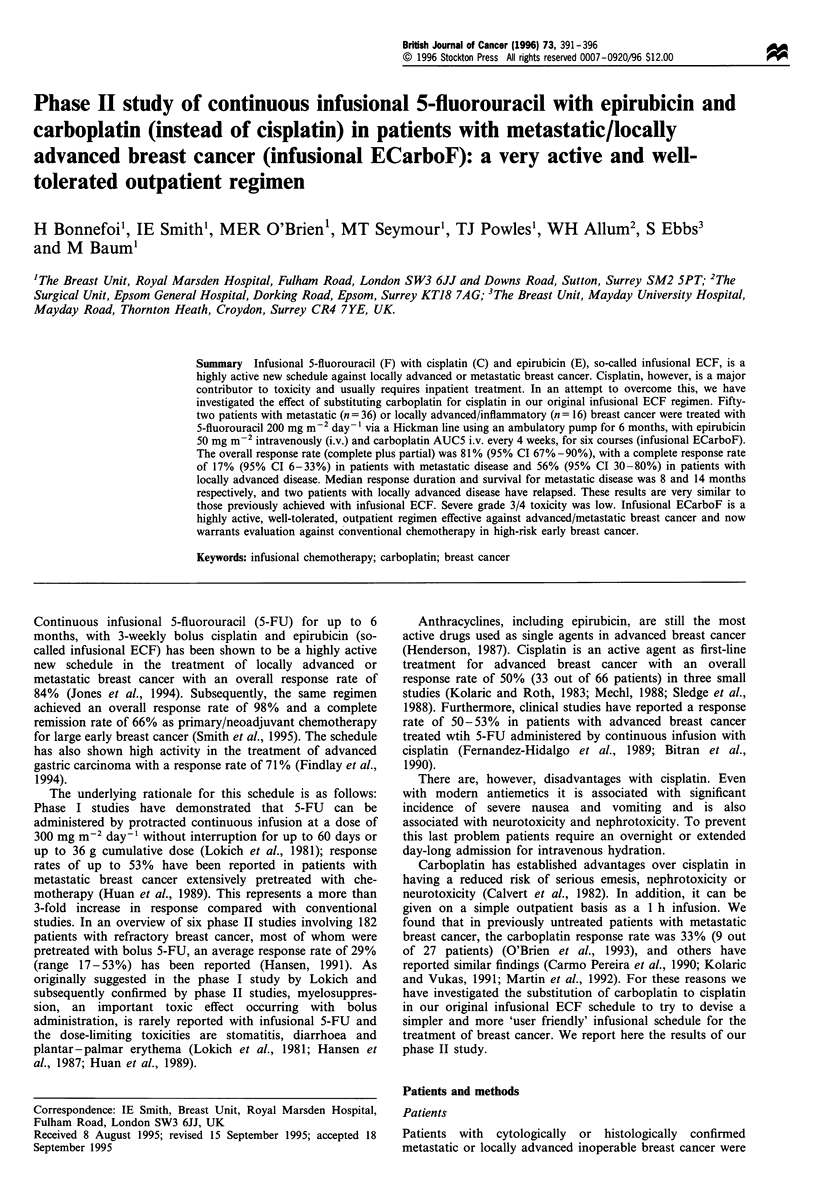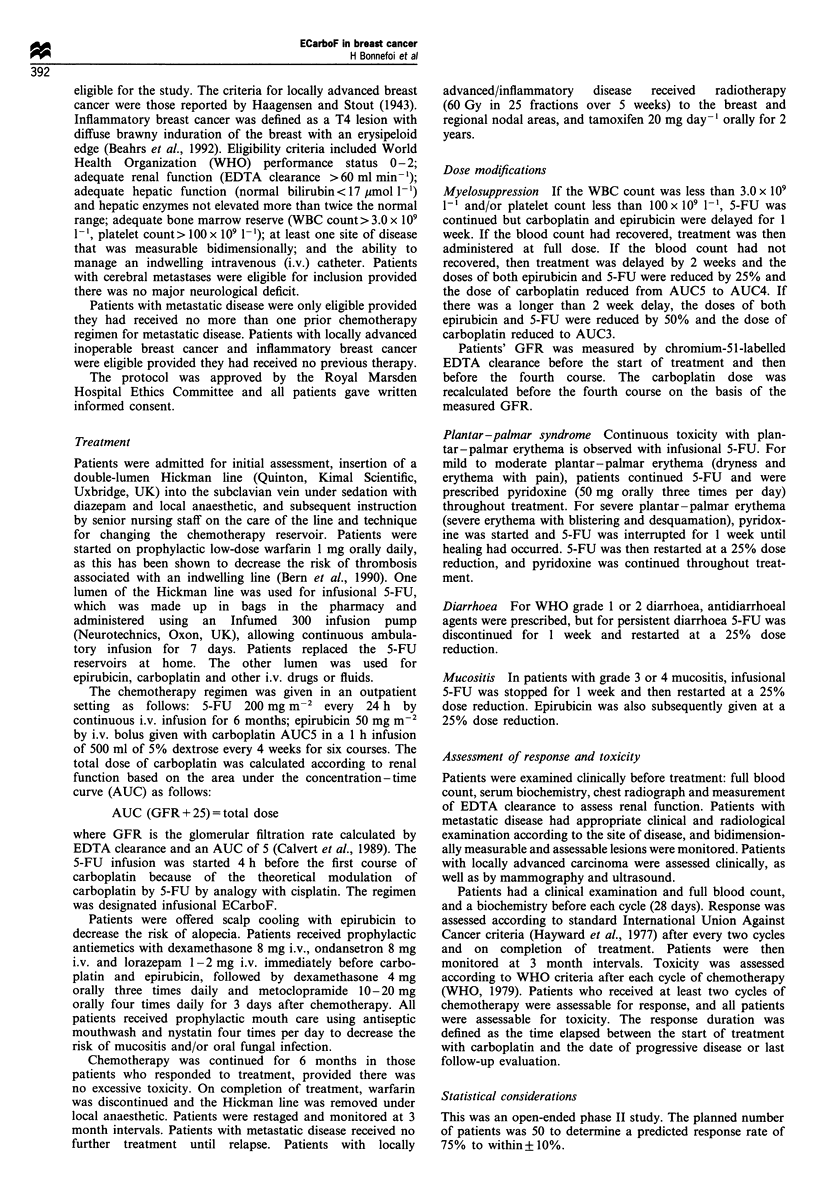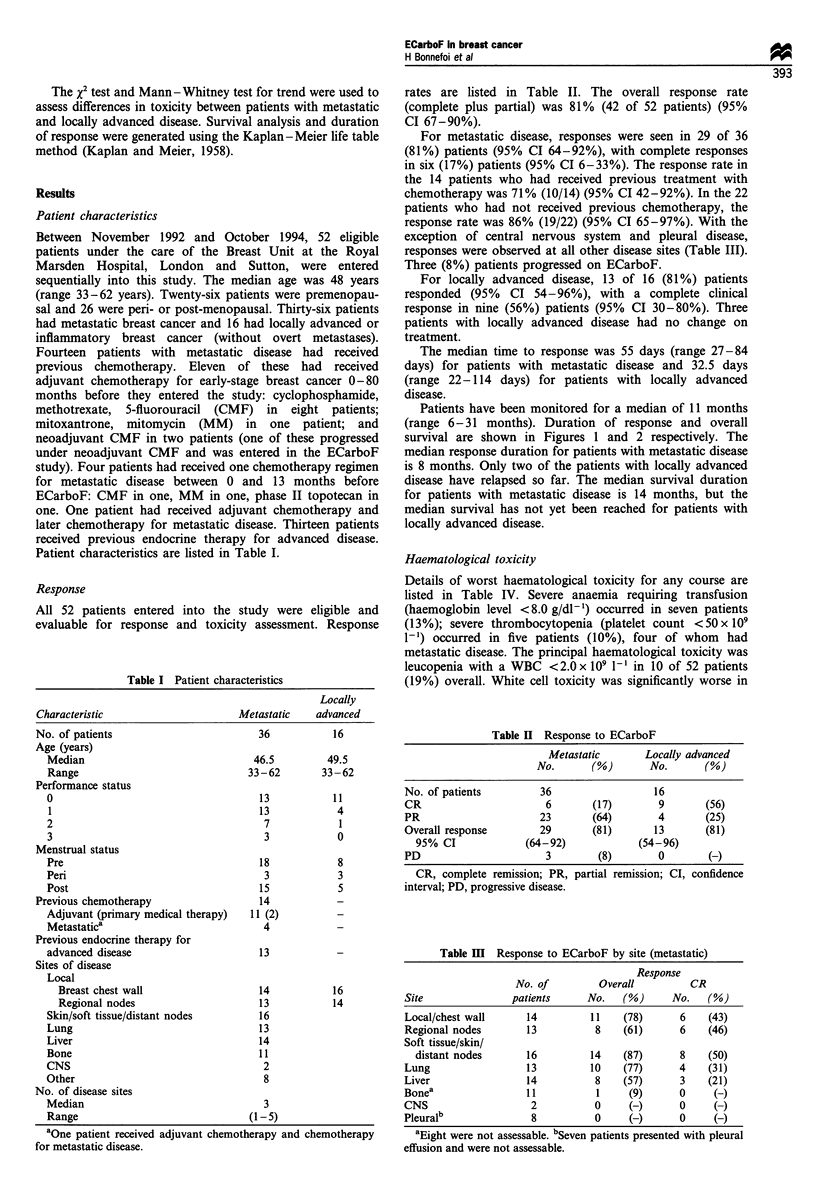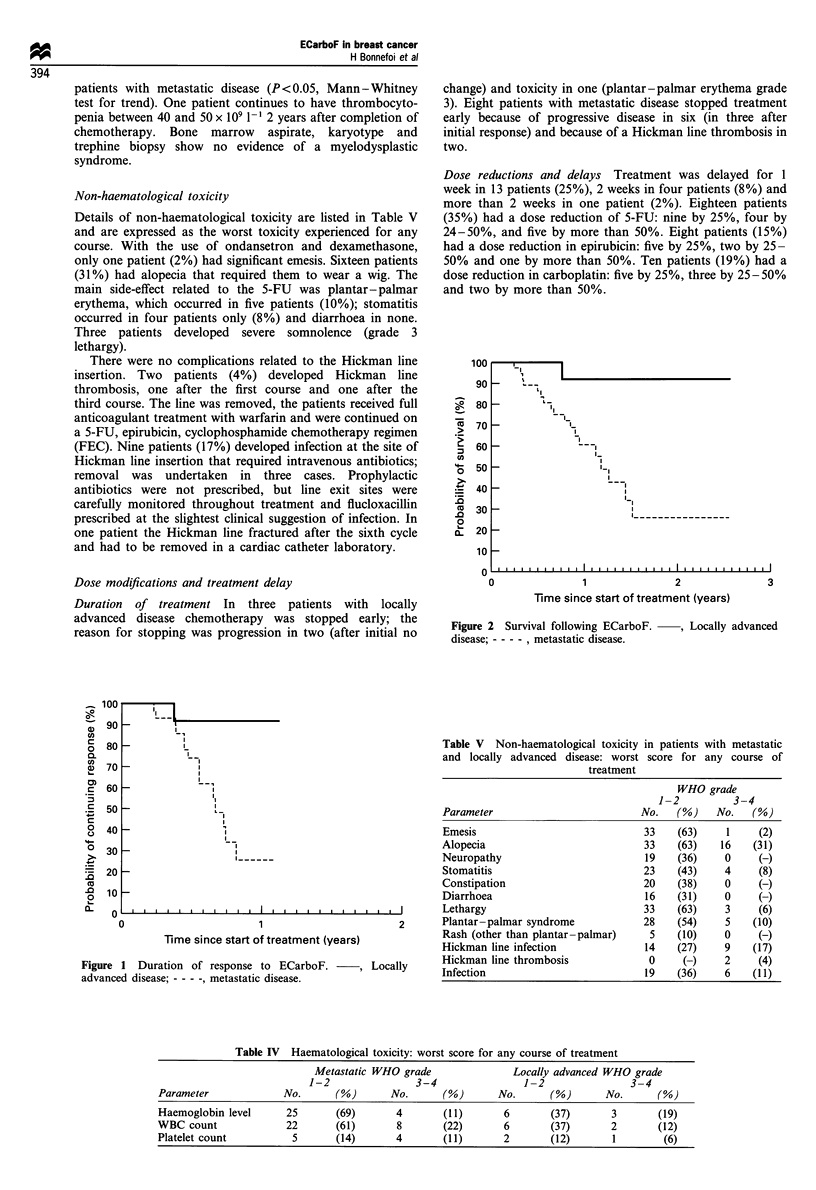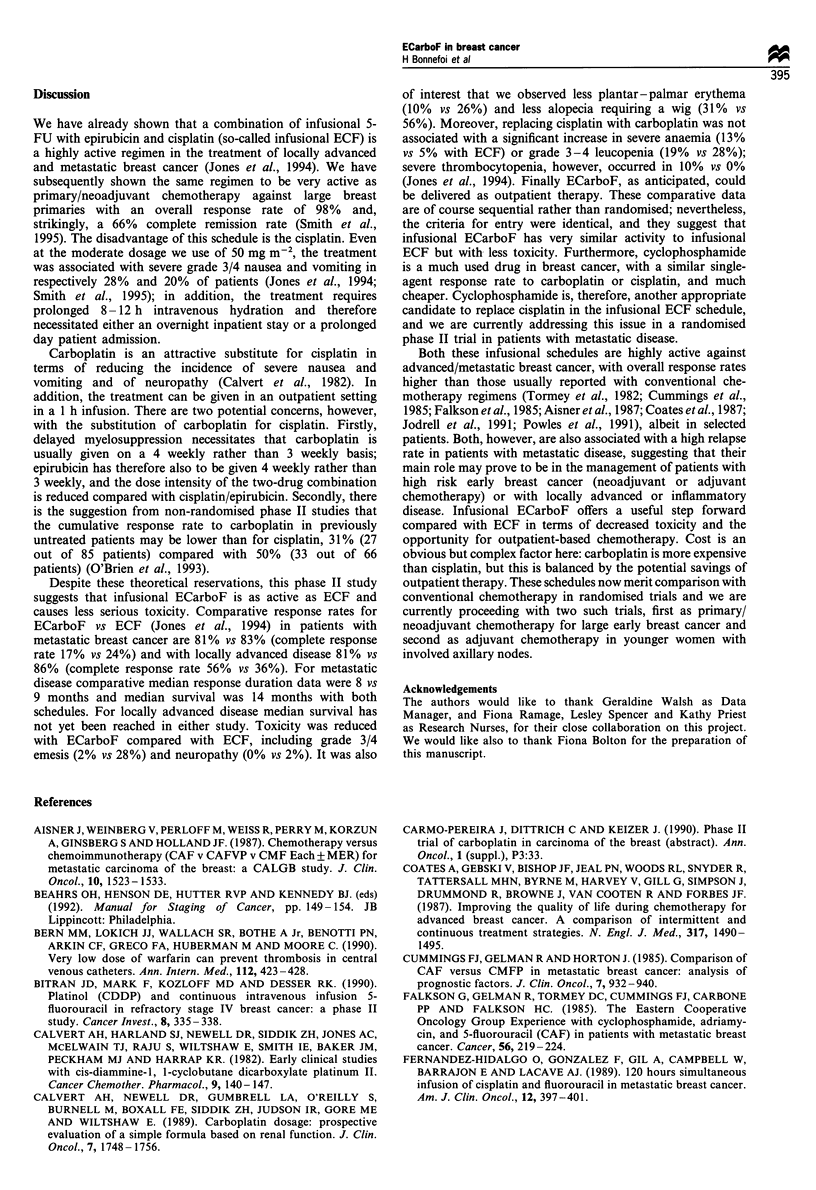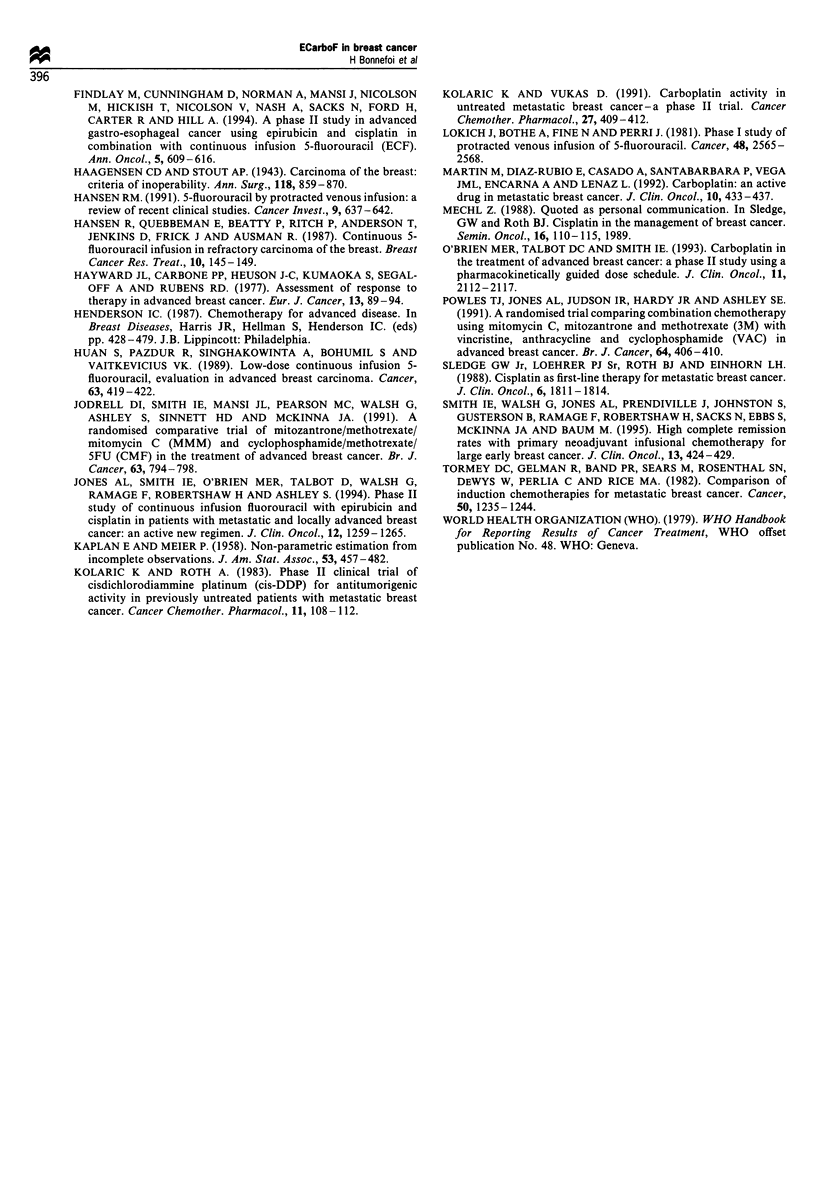# Phase II study of continuous infusional 5-fluorouracil with epirubicin and carboplatin (instead of cisplatin) in patients with metastatic/locally advanced breast cancer (infusional ECarboF): a very active and well-tolerated outpatient regimen.

**DOI:** 10.1038/bjc.1996.67

**Published:** 1996-02

**Authors:** H. Bonnefoi, I. E. Smith, M. E. O'Brien, M. T. Seymour, T. J. Powles, W. H. Allum, S. Ebbs, M. Baum

**Affiliations:** Breast Unit, Royal Marsden Hospital, London, UK.

## Abstract

Infusional 5-fluorouracil (F) with cisplatin (C) and epirubicin (E), so-called infusional ECF, is a highly active new schedule against locally advanced or metastatic breast cancer. Cisplatin, however, is a major contributor to toxicity and usually requires inpatient treatment. In an attempt to overcome this, we have investigated the effect of substituting carboplatin for cisplatin in our original infusional ECF regimen. Fifty-two patients with metastatic (n = 36) or locally advanced/inflammatory (n = 16) breast cancer were treated with 5-fluorouracil 200 mg m-2 day-1 via a Hickman line using an ambulatory pump for for 6 months, with epirubicin 50 mg m-2 intravenously (i.v.) and carboplatin AUC5 i.v. every 4 weeks, for six courses (infusional ECarboF). The overall response rate (complete plus partial) was 81% (95% CI 67%-90%), with a complete response rate of 17% (95% CI 6-33%) in patients with metastatic disease and 56% (95% CI 30-80%) in patients with locally advanced disease. Median response duration and survival for metastatic disease was 8 and 14 months respectively, and two patients with locally advanced disease have relapsed. These results are very similar to those previously achieved with infusional ECF. Severe grade 3/4 toxicity was low. Infusional ECarboF is a highly active, well-tolerated, outpatient regimen effective against advanced/metastatic breast cancer and now warrants evaluation against conventional chemotherapy in high-risk early breast cancer.


British Journal of Cancer (1996) 73, 391-396
© 1996 Stockton Press All rights reserved 0007-0920/96 $12.00

### Phase II study of continuous infusional 5-fluorouracil with epirubicin and carboplatin (instead of cisplatin) in patients with metastatic/locally advanced breast cancer (infusional ECarboF): a very active and well-tolerated outpatient regimen

H Bonnefoi[1], IE Smith[1], MER O'Brien[1], MT Seymour[1], TJ Powles[1], WH Allum[2], S Ebbs[3] and M Baum[1]

[1]*The Breast Unit, Royal Marsden Hospital, Fulham Road, London SW3 6JJ and Downs Road, Sutton, Surrey SM2 5PT;* [2]*The Surgical Unit, Epsom General Hospital, Dorking Road, Epsom, Surrey KT18 7AG;* [3]*The Breast Unit, Mayday University Hospital, Mayday Road, Thornton Heath, Croydon, Surrey CR4 7YE, UK.*

**Summary** Infusional 5-fluorouracil (F) with cisplatin (C) and epirubicin (E), so-called infusional ECF, is a highly active new schedule against locally advanced or metastatic breast cancer. Cisplatin, however, is a major contributor to toxicity and usually requires inpatient treatment. In an attempt to overcome this, we have investigated the effect of substituting carboplatin for cisplatin in our original infusional ECF regimen. Fifty-two patients with metastatic ($n=36$) or locally advanced/inflammatory ($n=16$) breast cancer were treated with 5-fluorouracil 200 mg $m^{-2}$ $day^{-1}$ via a Hickman line using an ambulatory pump for 6 months, with epirubicin 50 mg $m^{-2}$ intravenously (i.v.) and carboplatin AUC5 i.v. every 4 weeks, for six courses (infusional ECarboF). The overall response rate (complete plus partial) was 81% (95% CI 67%-90%), with a complete response rate of 17% (95% CI 6-33%) in patients with metastatic disease and 56% (95% CI 30-80%) in patients with locally advanced disease. Median response duration and survival for metastatic disease was 8 and 14 months respectively, and two patients with locally advanced disease have relapsed. These results are very similar to those previously achieved with infusional ECF. Severe grade 3/4 toxicity was low. Infusional ECarboF is a highly active, well-tolerated, outpatient regimen effective against advanced/metastatic breast cancer and now warrants evaluation against conventional chemotherapy in high-risk early breast cancer.

**Keywords:** infusional chemotherapy; carboplatin; breast cancer

Continuous infusional 5-fluorouracil (5-FU) for up to 6 months, with 3-weekly bolus cisplatin and epirubicin (so-called infusional ECF) has been shown to be a highly active new schedule in the treatment of locally advanced or metastatic breast cancer with an overall response rate of 84% (Jones *et al.*, 1994). Subsequently, the same regimen achieved an overall response rate of 98% and a complete remission rate of 66% as primary/neoadjuvant chemotherapy for large early breast cancer (Smith *et al.*, 1995). The schedule has also shown high activity in the treatment of advanced gastric carcinoma with a response rate of 71% (Findlay *et al.*, 1994).

The underlying rationale for this schedule is as follows: Phase I studies have demonstrated that 5-FU can be administered by protracted continuous infusion at a dose of 300 mg $m^{-2}$ $day^{-1}$ without interruption for up to 60 days or up to 36 g cumulative dose (Lokich *et al.*, 1981); response rates of up to 53% have been reported in patients with metastatic breast cancer extensively pretreated with chemotherapy (Huan *et al.*, 1989). This represents a more than 3-fold increase in response compared with conventional studies. In an overview of six phase II studies involving 182 patients with refractory breast cancer, most of whom were pretreated with bolus 5-FU, an average response rate of 29% (range 17-53%) has been reported (Hansen, 1991). As originally suggested in the phase I study by Lokich and subsequently confirmed by phase II studies, myelosuppression, an important toxic effect occurring with bolus administration, is rarely reported with infusional 5-FU and the dose-limiting toxicities are stomatitis, diarrhoea and plantar-palmar erythema (Lokich *et al.*, 1981; Hansen *et al.*, 1987; Huan *et al.*, 1989).

Correspondence: IE Smith, Breast Unit, Royal Marsden Hospital, Fulham Road, London SW3 6JJ, UK
Received 8 August 1995; revised 15 September 1995; accepted 18 September 1995

Anthracyclines, including epirubicin, are still the most active drugs used as single agents in advanced breast cancer (Henderson, 1987). Cisplatin is an active agent as first-line treatment for advanced breast cancer with an overall response rate of 50% (33 out of 66 patients) in three small studies (Kolaric and Roth, 1983; Mechl, 1988; Sledge *et al.*, 1988). Furthermore, clinical studies have reported a response rate of 50-53% in patients with advanced breast cancer treated wtih 5-FU administered by continuous infusion with cisplatin (Fernandez-Hidalgo *et al.*, 1989; Bitran *et al.*, 1990).

There are, however, disadvantages with cisplatin. Even with modern antiemetics it is associated with significant incidence of severe nausea and vomiting and is also associated with neurotoxicity and nephrotoxicity. To prevent this last problem patients require an overnight or extended day-long admission for intravenous hydration.

Carboplatin has established advantages over cisplatin in having a reduced risk of serious emesis, nephrotoxicity or neurotoxicity (Calvert *et al.*, 1982). In addition, it can be given on a simple outpatient basis as a 1 h infusion. We found that in previously untreated patients with metastatic breast cancer, the carboplatin response rate was 33% (9 out of 27 patients) (O'Brien *et al.*, 1993), and others have reported similar findings (Carmo Pereira *et al.*, 1990; Kolaric and Vukas, 1991; Martin *et al.*, 1992). For these reasons we have investigated the substitution of carboplatin to cisplatin in our original infusional ECF schedule to try to devise a simpler and more 'user friendly' infusional schedule for the treatment of breast cancer. We report here the results of our phase II study.

### Patients and methods

### *Patients*

Patients with cytologically or histologically confirmed metastatic or locally advanced inoperable breast cancer were eligible for the study. The criteria for locally advanced breast cancer were those reported by Haagensen and Stout (1943). Inflammatory breast cancer was defined as a T4 lesion with diffuse brawny induration of the breast with an erysipeloid edge (Beahrs et al., 1992). Eligibility criteria included World Health Organization (WHO) performance status 0–2; adequate renal function (EDTA clearance $>60$ ml $min^{-1}$); adequate hepatic function (normal bilirubin $<17$ $\mu mol\ l^{-1}$) and hepatic enzymes not elevated more than twice the normal range; adequate bone marrow reserve (WBC count $>3.0\times10^9$ $l^{-1}$, platelet count $>100\times10^9$ $l^{-1}$); at least one site of disease that was measurable bidimensionally; and the ability to manage an indwelling intravenous (i.v.) catheter. Patients with cerebral metastases were eligible for inclusion provided there was no major neurological deficit.

Patients with metastatic disease were only eligible provided they had received no more than one prior chemotherapy regimen for metastatic disease. Patients with locally advanced inoperable breast cancer and inflammatory breast cancer were eligible provided they had received no previous therapy.

The protocol was approved by the Royal Marsden Hospital Ethics Committee and all patients gave written informed consent.

### Treatment

Patients were admitted for initial assessment, insertion of a double-lumen Hickman line (Quinton, Kimal Scientific, Uxbridge, UK) into the subclavian vein under sedation with diazepam and local anaesthetic, and subsequent instruction by senior nursing staff on the care of the line and technique for changing the chemotherapy reservoir. Patients were started on prophylactic low-dose warfarin 1 mg orally daily, as this has been shown to decrease the risk of thrombosis associated with an indwelling line (Bern et al., 1990). One lumen of the Hickman line was used for infusional 5-FU, which was made up in bags in the pharmacy and administered using an Infumed 300 infusion pump (Neurotechnics, Oxon, UK), allowing continuous ambulatory infusion for 7 days. Patients replaced the 5-FU reservoirs at home. The other lumen was used for epirubicin, carboplatin and other i.v. drugs or fluids.

The chemotherapy regimen was given in an outpatient setting as follows: 5-FU 200 mg $m^{-2}$ every 24 h by continuous i.v. infusion for 6 months; epirubicin 50 mg $m^{-2}$ by i.v. bolus given with carboplatin AUC5 in a 1 h infusion of 500 ml of 5% dextrose every 4 weeks for six courses. The total dose of carboplatin was calculated according to renal function based on the area under the concentration–time curve (AUC) as follows:

$$\text{AUC (GFR} + 25) = \text{total dose}$$

where GFR is the glomerular filtration rate calculated by EDTA clearance and an AUC of 5 (Calvert et al., 1989). The 5-FU infusion was started 4 h before the first course of carboplatin because of the theoretical modulation of carboplatin by 5-FU by analogy with cisplatin. The regimen was designated infusional ECarboF.

Patients were offered scalp cooling with epirubicin to decrease the risk of alopecia. Patients received prophylactic antiemetics with dexamethasone 8 mg i.v., ondansetron 8 mg i.v. and lorazepam 1–2 mg i.v. immediately before carboplatin and epirubicin, followed by dexamethasone 4 mg orally three times daily and metoclopramide 10–20 mg orally four times daily for 3 days after chemotherapy. All patients received prophylactic mouth care using antiseptic mouthwash and nystatin four times per day to decrease the risk of mucositis and/or oral fungal infection.

Chemotherapy was continued for 6 months in those patients who responded to treatment, provided there was no excessive toxicity. On completion of treatment, warfarin was discontinued and the Hickman line was removed under local anaesthetic. Patients were restaged and monitored at 3 month intervals. Patients with metastatic disease received no further treatment until relapse. Patients with locally advanced/inflammatory disease received radiotherapy (60 Gy in 25 fractions over 5 weeks) to the breast and regional nodal areas, and tamoxifen 20 mg $day^{-1}$ orally for 2 years.

### Dose modifications

*Myelosuppression* If the WBC count was less than $3.0\times10^9$ $l^{-1}$ and/or platelet count less than $100\times10^9$ $l^{-1}$, 5-FU was continued but carboplatin and epirubicin were delayed for 1 week. If the blood count had recovered, treatment was then administered at full dose. If the blood count had not recovered, then treatment was delayed by 2 weeks and the doses of both epirubicin and 5-FU were reduced by 25% and the dose of carboplatin reduced from AUC5 to AUC4. If there was a longer than 2 week delay, the doses of both epirubicin and 5-FU were reduced by 50% and the dose of carboplatin reduced to AUC3.

Patients' GFR was measured by chromium-51-labelled EDTA clearance before the start of treatment and then before the fourth course. The carboplatin dose was recalculated before the fourth course on the basis of the measured GFR.

*Plantar–palmar syndrome* Continuous toxicity with plantar–palmar erythema is observed with infusional 5-FU. For mild to moderate plantar–palmar erythema (dryness and erythema with pain), patients continued 5-FU and were prescribed pyridoxine (50 mg orally three times per day) throughout treatment. For severe plantar–palmar erythema (severe erythema with blistering and desquamation), pyridoxine was started and 5-FU was interrupted for 1 week until healing had occurred. 5-FU was then restarted at a 25% dose reduction, and pyridoxine was continued throughout treatment.

*Diarrhoea* For WHO grade 1 or 2 diarrhoea, antidiarrhoeal agents were prescribed, but for persistent diarrhoea 5-FU was discontinued for 1 week and restarted at a 25% dose reduction.

*Mucositis* In patients with grade 3 or 4 mucositis, infusional 5-FU was stopped for 1 week and then restarted at a 25% dose reduction. Epirubicin was also subsequently given at a 25% dose reduction.

### Assessment of response and toxicity

Patients were examined clinically before treatment: full blood count, serum biochemistry, chest radiograph and measurement of EDTA clearance to assess renal function. Patients with metastatic disease had appropriate clinical and radiological examination according to the site of disease, and bidimensionally measurable and assessable lesions were monitored. Patients with locally advanced carcinoma were assessed clinically, as well as by mammography and ultrasound.

Patients had a clinical examination and full blood count, and a biochemistry before each cycle (28 days). Response was assessed according to standard International Union Against Cancer criteria (Hayward et al., 1977) after every two cycles and on completion of treatment. Patients were then monitored at 3 month intervals. Toxicity was assessed according to WHO criteria after each cycle of chemotherapy (WHO, 1979). Patients who received at least two cycles of chemotherapy were assessable for response, and all patients were assessable for toxicity. The response duration was defined as the time elapsed between the start of treatment with carboplatin and the date of progressive disease or last follow-up evaluation.

### Statistical considerations

This was an open-ended phase II study. The planned number of patients was 50 to determine a predicted response rate of 75% to within $\pm10\%$.

The $\chi^2$ test and Mann–Whitney test for trend were used to assess differences in toxicity between patients with metastatic and locally advanced disease. Survival analysis and duration of response were generated using the Kaplan–Meier life table method (Kaplan and Meier, 1958).

### Results

### *Patient characteristics*

Between November 1992 and October 1994, 52 eligible patients under the care of the Breast Unit at the Royal Marsden Hospital, London and Sutton, were entered sequentially into this study. The median age was 48 years (range 33–62 years). Twenty-six patients were premenopausal and 26 were peri- or post-menopausal. Thirty-six patients had metastatic breast cancer and 16 had locally advanced or inflammatory breast cancer (without overt metastases). Fourteen patients with metastatic disease had received previous chemotherapy. Eleven of these had received adjuvant chemotherapy for early-stage breast cancer 0–80 months before they entered the study: cyclophosphamide, methotrexate, 5-fluorouracil (CMF) in eight patients; mitoxantrone, mitomycin (MM) in one patient; and neoadjuvant CMF in two patients (one of these progressed under neoadjuvant CMF and was entered in the ECarboF study). Four patients had received one chemotherapy regimen for metastatic disease between 0 and 13 months before ECarboF: CMF in one, MM in one, phase II topotecan in one. One patient had received adjuvant chemotherapy and later chemotherapy for metastatic disease. Thirteen patients received previous endocrine therapy for advanced disease. Patient characteristics are listed in Table I.

### *Response*

All 52 patients entered into the study were eligible and evaluable for response and toxicity assessment. Response rates are listed in Table II. The overall response rate (complete plus partial) was 81% (42 of 52 patients) (95% CI 67–90%).

For metastatic disease, responses were seen in 29 of 36 (81%) patients (95% CI 64–92%), with complete responses in six (17%) patients (95% CI 6–33%). The response rate in the 14 patients who had received previous treatment with chemotherapy was 71% (10/14) (95% CI 42–92%). In the 22 patients who had not received previous chemotherapy, the response rate was 86% (19/22) (95% CI 65–97%). With the exception of central nervous system and pleural disease, responses were observed at all other disease sites (Table III). Three (8%) patients progressed on ECarboF.

For locally advanced disease, 13 of 16 (81%) patients responded (95% CI 54–96%), with a complete clinical response in nine (56%) patients (95% CI 30–80%). Three patients with locally advanced disease had no change on treatment.

The median time to response was 55 days (range 27–84 days) for patients with metastatic disease and 32.5 days (range 22–114 days) for patients with locally advanced disease.

Patients have been monitored for a median of 11 months (range 6–31 months). Duration of response and overall survival are shown in Figures 1 and 2 respectively. The median response duration for patients with metastatic disease is 8 months. Only two of the patients with locally advanced disease have relapsed so far. The median survival duration for patients with metastatic disease is 14 months, but the median survival has not yet been reached for patients with locally advanced disease.

### *Haematological toxicity*

Details of worst haematological toxicity for any course are listed in Table IV. Severe anaemia requiring transfusion (haemoglobin level $<8.0\ \text{g/dl}^{-1}$) occurred in seven patients (13%); severe thrombocytopenia (platelet count $<50\times10^9\ \text{l}^{-1}$) occurred in five patients (10%), four of whom had metastatic disease. The principal haematological toxicity was leucopenia with a WBC $<2.0\times10^9\ \text{l}^{-1}$ in 10 of 52 patients (19%) overall. White cell toxicity was significantly worse in

**Table I** Patient characteristics

| *Characteristic* | *Metastatic* | *Locally advanced* |
|---|---|---|
| No. of patients | 36 | 16 |
| Age (years) | | |
| Median | 46.5 | 49.5 |
| Range | 33–62 | 33–62 |
| Performance status | | |
| 0 | 13 | 11 |
| 1 | 13 | 4 |
| 2 | 7 | 1 |
| 3 | 3 | 0 |
| Menstrual status | | |
| Pre | 18 | 8 |
| Peri | 3 | 3 |
| Post | 15 | 5 |
| Previous chemotherapy | 14 | – |
| Adjuvant (primary medical therapy) | 11 (2) | – |
| Metastatic[a] | 4 | – |
| Previous endocrine therapy for advanced disease | 13 | – |
| Sites of disease | | |
| Local | | |
| Breast chest wall | 14 | 16 |
| Regional nodes | 13 | 14 |
| Skin/soft tissue/distant nodes | 16 | |
| Lung | 13 | |
| Liver | 14 | |
| Bone | 11 | |
| CNS | 2 | |
| Other | 8 | |
| No. of disease sites | | |
| Median | 3 | |
| Range | (1–5) | |

[a]One patient received adjuvant chemotherapy and chemotherapy for metastatic disease.

**Table II** Response to ECarboF

| | *Metastatic No.* | *(%)* | *Locally advanced No.* | *(%)* |
|---|---|---|---|---|
| No. of patients | 36 | | 16 | |
| CR | 6 | (17) | 9 | (56) |
| PR | 23 | (64) | 4 | (25) |
| Overall response | 29 | (81) | 13 | (81) |
| 95% CI | (64–92) | | (54–96) | |
| PD | 3 | (8) | 0 | (–) |

CR, complete remission; PR, partial remission; CI, confidence interval; PD, progressive disease.

**Table III** Response to ECarboF by site (metastatic)

| *Site* | *No. of patients* | *Response Overall No.* | *(%)* | *CR No.* | *(%)* |
|---|---|---|---|---|---|
| Local/chest wall | 14 | 11 | (78) | 6 | (43) |
| Regional nodes | 13 | 8 | (61) | 6 | (46) |
| Soft tissue/skin/ distant nodes | 16 | 14 | (87) | 8 | (50) |
| Lung | 13 | 10 | (77) | 4 | (31) |
| Liver | 14 | 8 | (57) | 3 | (21) |
| Bone[a] | 11 | 1 | (9) | 0 | (–) |
| CNS | 2 | 0 | (–) | 0 | (–) |
| Pleural[b] | 8 | 0 | (–) | 0 | (–) |

[a]Eight were not assessable. [b]Seven patients presented with pleural effusion and were not assessable.

patients with metastatic disease ($P<0.05$, Mann–Whitney test for trend). One patient continues to have thrombocytopenia between 40 and $50\times10^9\ l^{-1}$ 2 years after completion of chemotherapy. Bone marrow aspirate, karyotype and trephine biopsy show no evidence of a myelodysplastic syndrome.

### *Non-haematological toxicity*

Details of non-haematological toxicity are listed in Table V and are expressed as the worst toxicity experienced for any course. With the use of ondansetron and dexamethasone, only one patient (2%) had significant emesis. Sixteen patients (31%) had alopecia that required them to wear a wig. The main side-effect related to the 5-FU was plantar–palmar erythema, which occurred in five patients (10%); stomatitis occurred in four patients only (8%) and diarrhoea in none. Three patients developed severe somnolence (grade 3 lethargy).

There were no complications related to the Hickman line insertion. Two patients (4%) developed Hickman line thrombosis, one after the first course and one after the third course. The line was removed, the patients received full anticoagulant treatment with warfarin and were continued on a 5-FU, epirubicin, cyclophosphamide chemotherapy regimen (FEC). Nine patients (17%) developed infection at the site of Hickman line insertion that required intravenous antibiotics; removal was undertaken in three cases. Prophylactic antibiotics were not prescribed, but line exit sites were carefully monitored throughout treatment and flucloxacillin prescribed at the slightest clinical suggestion of infection. In one patient the Hickman line fractured after the sixth cycle and had to be removed in a cardiac catheter laboratory.

### *Dose modifications and treatment delay*

*Duration of treatment* In three patients with locally advanced disease chemotherapy was stopped early; the reason for stopping was progression in two (after initial no change) and toxicity in one (plantar–palmar erythema grade 3). Eight patients with metastatic disease stopped treatment early because of progressive disease in six (in three after initial response) and because of a Hickman line thrombosis in two.

*Dose reductions and delays* Treatment was delayed for 1 week in 13 patients (25%), 2 weeks in four patients (8%) and more than 2 weeks in one patient (2%). Eighteen patients (35%) had a dose reduction of 5-FU: nine by 25%, four by 24–50%, and five by more than 50%. Eight patients (15%) had a dose reduction in epirubicin: five by 25%, two by 25–50% and one by more than 50%. Ten patients (19%) had a dose reduction in carboplatin: five by 25%, three by 25–50% and two by more than 50%.

**Figure 2** Survival following ECarboF. ——, Locally advanced disease; - - - - , metastatic disease.

**Figure 1** Duration of response to ECarboF. ——, Locally advanced disease; - - - -, metastatic disease.

**Table V** Non-haematological toxicity in patients with metastatic and locally advanced disease: worst score for any course of treatment

| *Parameter* | *WHO grade 1–2 No.* | *(%)* | *3–4 No.* | *(%)* |
|---|---|---|---|---|
| Emesis | 33 | (63) | 1 | (2) |
| Alopecia | 33 | (63) | 16 | (31) |
| Neuropathy | 19 | (36) | 0 | (–) |
| Stomatitis | 23 | (43) | 4 | (8) |
| Constipation | 20 | (38) | 0 | (–) |
| Diarrhoea | 16 | (31) | 0 | (–) |
| Lethargy | 33 | (63) | 3 | (6) |
| Plantar–palmar syndrome | 28 | (54) | 5 | (10) |
| Rash (other than plantar–palmar) | 5 | (10) | 0 | (–) |
| Hickman line infection | 14 | (27) | 9 | (17) |
| Hickman line thrombosis | 0 | (–) | 2 | (4) |
| Infection | 19 | (36) | 6 | (11) |

**Table IV** Haematological toxicity: worst score for any course of treatment

| *Parameter* | *Metastatic WHO grade 1–2 No.* | *(%)* | *3–4 No.* | *(%)* | *Locally advanced WHO grade 1–2 No.* | *(%)* | *3–4 No.* | *(%)* |
|---|---|---|---|---|---|---|---|---|
| Haemoglobin level | 25 | (69) | 4 | (11) | 6 | (37) | 3 | (19) |
| WBC count | 22 | (61) | 8 | (22) | 6 | (37) | 2 | (12) |
| Platelet count | 5 | (14) | 4 | (11) | 2 | (12) | 1 | (6) |

### Discussion

We have already shown that a combination of infusional 5-FU with epirubicin and cisplatin (so-called infusional ECF) is a highly active regimen in the treatment of locally advanced and metastatic breast cancer (Jones *et al.*, 1994). We have subsequently shown the same regimen to be very active as primary/neoadjuvant chemotherapy against large breast primaries with an overall response rate of 98% and, strikingly, a 66% complete remission rate (Smith *et al.*, 1995). The disadvantage of this schedule is the cisplatin. Even at the moderate dosage we use of 50 mg $m^{-2}$, the treatment was associated with severe grade 3/4 nausea and vomiting in respectively 28% and 20% of patients (Jones *et al.*, 1994; Smith *et al.*, 1995); in addition, the treatment requires prolonged 8–12 h intravenous hydration and therefore necessitated either an overnight inpatient stay or a prolonged day patient admission.

Carboplatin is an attractive substitute for cisplatin in terms of reducing the incidence of severe nausea and vomiting and of neuropathy (Calvert *et al.*, 1982). In addition, the treatment can be given in an outpatient setting in a 1 h infusion. There are two potential concerns, however, with the substitution of carboplatin for cisplatin. Firstly, delayed myelosuppression necessitates that carboplatin is usually given on a 4 weekly rather than 3 weekly basis; epirubicin has therefore also to be given 4 weekly rather than 3 weekly, and the dose intensity of the two-drug combination is reduced compared with cisplatin/epirubicin. Secondly, there is the suggestion from non-randomised phase II studies that the cumulative response rate to carboplatin in previously untreated patients may be lower than for cisplatin, 31% (27 out of 85 patients) compared with 50% (33 out of 66 patients) (O'Brien *et al.*, 1993).

Despite these theoretical reservations, this phase II study suggests that infusional ECarboF is as active as ECF and causes less serious toxicity. Comparative response rates for ECarboF *vs* ECF (Jones *et al.*, 1994) in patients with metastatic breast cancer are 81% *vs* 83% (complete response rate 17% *vs* 24%) and with locally advanced disease 81% *vs* 86% (complete response rate 56% *vs* 36%). For metastatic disease comparative median response duration data were 8 *vs* 9 months and median survival was 14 months with both schedules. For locally advanced disease median survival has not yet been reached in either study. Toxicity was reduced with ECarboF compared with ECF, including grade 3/4 emesis (2% *vs* 28%) and neuropathy (0% *vs* 2%). It was also of interest that we observed less plantar–palmar erythema (10% *vs* 26%) and less alopecia requiring a wig (31% *vs* 56%). Moreover, replacing cisplatin with carboplatin was not associated with a significant increase in severe anaemia (13% *vs* 5% with ECF) or grade 3–4 leucopenia (19% *vs* 28%); severe thrombocytopenia, however, occurred in 10% *vs* 0% (Jones *et al.*, 1994). Finally ECarboF, as anticipated, could be delivered as outpatient therapy. These comparative data are of course sequential rather than randomised; nevertheless, the criteria for entry were identical, and they suggest that infusional ECarboF has very similar activity to infusional ECF but with less toxicity. Furthermore, cyclophosphamide is a much used drug in breast cancer, with a similar single-agent response rate to carboplatin or cisplatin, and much cheaper. Cyclophosphamide is, therefore, another appropriate candidate to replace cisplatin in the infusional ECF schedule, and we are currently addressing this issue in a randomised phase II trial in patients with metastatic disease.

Both these infusional schedules are highly active against advanced/metastatic breast cancer, with overall response rates higher than those usually reported with conventional chemotherapy regimens (Tormey *et al.*, 1982; Cummings *et al.*, 1985; Falkson *et al.*, 1985; Aisner *et al.*, 1987; Coates *et al.*, 1987; Jodrell *et al.*, 1991; Powles *et al.*, 1991), albeit in selected patients. Both, however, are also associated with a high relapse rate in patients with metastatic disease, suggesting that their main role may prove to be in the management of patients with high risk early breast cancer (neoadjuvant or adjuvant chemotherapy) or with locally advanced or inflammatory disease. Infusional ECarboF offers a useful step forward compared with ECF in terms of decreased toxicity and the opportunity for outpatient-based chemotherapy. Cost is an obvious but complex factor here: carboplatin is more expensive than cisplatin, but this is balanced by the potential savings of outpatient therapy. These schedules now merit comparison with conventional chemotherapy in randomised trials and we are currently proceeding with two such trials, first as primary/neoadjuvant chemotherapy for large early breast cancer and second as adjuvant chemotherapy in younger women with involved axillary nodes.

**Acknowledgements**
The authors would like to thank Geraldine Walsh as Data Manager, and Fiona Ramage, Lesley Spencer and Kathy Priest as Research Nurses, for their close collaboration on this project. We would like also to thank Fiona Bolton for the preparation of this manuscript.

### References

AISNER J, WEINBERG V, PERLOFF M, WEISS R, PERRY M, KORZUN A, GINSBERG S AND HOLLAND JF. (1987). Chemotherapy versus chemoimmunotherapy (CAF v CAFVP v CMF Each ± MER) for metastatic carcinoma of the breast: a CALGB study. *J. Clin. Oncol.*, **10**, 1523–1533.

BEAHRS OH, HENSON DE, HUTTER RVP AND KENNEDY BJ. (eds) (1992). *Manual for Staging of Cancer*, pp. 149–154. JB Lippincott: Philadelphia.

BERN MM, LOKICH JJ, WALLACH SR, BOTHE A Jr, BENOTTI PN, ARKIN CF, GRECO FA, HUBERMAN M AND MOORE C. (1990). Very low dose of warfarin can prevent thrombosis in central venous catheters. *Ann. Intern. Med.*, **112**, 423–428.

BITRAN JD, MARK F, KOZLOFF MD AND DESSER RK. (1990). Platinol (CDDP) and continuous intravenous infusion 5-fluorouracil in refractory stage IV breast cancer: a phase II study. *Cancer Invest.*, **8**, 335–338.

CALVERT AH, HARLAND SJ, NEWELL DR, SIDDIK ZH, JONES AC, MCELWAIN TJ, RAJU S, WILTSHAW E, SMITH IE, BAKER JM, PECKHAM MJ AND HARRAP KR. (1982). Early clinical studies with cis-diammine-1, 1-cyclobutane dicarboxylate platinum II. *Cancer Chemother. Pharmacol.*, **9**, 140–147.

CALVERT AH, NEWELL DR, GUMBRELL LA, O'REILLY S, BURNELL M, BOXALL FE, SIDDIK ZH, JUDSON IR, GORE ME AND WILTSHAW E. (1989). Carboplatin dosage: prospective evaluation of a simple formula based on renal function. *J. Clin. Oncol.*, **7**, 1748–1756.

CARMO-PEREIRA J, DITTRICH C AND KEIZER J. (1990). Phase II trial of carboplatin in carcinoma of the breast (abstract). *Ann. Oncol.*, **1** (suppl.), P3:33.

COATES A, GEBSKI V, BISHOP JF, JEAL PN, WOODS RL, SNYDER R, TATTERSALL MHN, BYRNE M, HARVEY V, GILL G, SIMPSON J, DRUMMOND R, BROWNE J, VAN COOTEN R AND FORBES JF. (1987). Improving the quality of life during chemotherapy for advanced breast cancer. A comparison of intermittent and continuous treatment strategies. *N. Engl. J. Med.*, **317**, 1490–1495.

CUMMINGS FJ, GELMAN R AND HORTON J. (1985). Comparison of CAF versus CMFP in metastatic breast cancer: analysis of prognostic factors. *J. Clin. Oncol.*, **7**, 932–940.

FALKSON G, GELMAN R, TORMEY DC, CUMMINGS FJ, CARBONE PP AND FALKSON HC. (1985). The Eastern Cooperative Oncology Group Experience with cyclophosphamide, adriamycin, and 5-fluorouracil (CAF) in patients with metastatic breast cancer. *Cancer*, **56**, 219–224.

FERNANDEZ-HIDALGO O, GONZALEZ F, GIL A, CAMPBELL W, BARRAJON E AND LACAVE AJ. (1989). 120 hours simultaneous infusion of cisplatin and fluorouracil in metastatic breast cancer. *Am. J. Clin. Oncol.*, **12**, 397–401.

FINDLAY M, CUNNINGHAM D, NORMAN A, MANSI J, NICOLSON M, HICKISH T, NICOLSON V, NASH A, SACKS N, FORD H, CARTER R AND HILL A. (1994). A phase II study in advanced gastro-esophageal cancer using epirubicin and cisplatin in combination with continuous infusion 5-fluorouracil (ECF). *Ann. Oncol.*, **5**, 609-616.

HAAGENSEN CD AND STOUT AP. (1943). Carcinoma of the breast: criteria of inoperability. *Ann. Surg.*, **118**, 859-870.

HANSEN RM. (1991). 5-fluorouracil by protracted venous infusion: a review of recent clinical studies. *Cancer Invest.*, **9**, 637-642.

HANSEN R, QUEBBEMAN E, BEATTY P, RITCH P, ANDERSON T, JENKINS D, FRICK J AND AUSMAN R. (1987). Continuous 5-fluorouracil infusion in refractory carcinoma of the breast. *Breast Cancer Res. Treat.*, **10**, 145-149.

HAYWARD JL, CARBONE PP, HEUSON J-C, KUMAOKA S, SEGALOFF A AND RUBENS RD. (1977). Assessment of response to therapy in advanced breast cancer. *Eur. J. Cancer*, **13**, 89-94.

HENDERSON IC. (1987). Chemotherapy for advanced disease. In *Breast Diseases*, Harris JR, Hellman S, Henderson IC. (eds) pp. 428-479. J.B. Lippincott: Philadelphia.

HUAN S, PAZDUR R, SINGHAKOWINTA A, BOHUMIL S AND VAITKEVICIUS VK. (1989). Low-dose continuous infusion 5-fluorouracil, evaluation in advanced breast carcinoma. *Cancer*, **63**, 419-422.

JODRELL DI, SMITH IE, MANSI JL, PEARSON MC, WALSH G, ASHLEY S, SINNETT HD AND MCKINNA JA. (1991). A randomised comparative trial of mitozantrone/methotrexate/mitomycin C (MMM) and cyclophosphamide/methotrexate/5FU (CMF) in the treatment of advanced breast cancer. *Br. J. Cancer*, **63**, 794-798.

JONES AL, SMITH IE, O'BRIEN MER, TALBOT D, WALSH G, RAMAGE F, ROBERTSHAW H AND ASHLEY S. (1994). Phase II study of continuous infusion fluorouracil with epirubicin and cisplatin in patients with metastatic and locally advanced breast cancer: an active new regimen. *J. Clin. Oncol.*, **12**, 1259-1265.

KAPLAN E AND MEIER P. (1958). Non-parametric estimation from incomplete observations. *J. Am. Stat. Assoc.*, **53**, 457-482.

KOLARIC K AND ROTH A. (1983). Phase II clinical trial of cisdichlorodiammine platinum (cis-DDP) for antitumorigenic activity in previously untreated patients with metastatic breast cancer. *Cancer Chemother. Pharmacol.*, **11**, 108-112.

KOLARIC K AND VUKAS D. (1991). Carboplatin activity in untreated metastatic breast cancer-a phase II trial. *Cancer Chemother. Pharmacol.*, **27**, 409-412.

LOKICH J, BOTHE A, FINE N AND PERRI J. (1981). Phase I study of protracted venous infusion of 5-fluorouracil. *Cancer*, **48**, 2565-2568.

MARTIN M, DIAZ-RUBIO E, CASADO A, SANTABARBARA P, VEGA JML, ENCARNA A AND LENAZ L. (1992). Carboplatin: an active drug in metastatic breast cancer. *J. Clin. Oncol.*, **10**, 433-437.

MECHL Z. (1988). Quoted as personal communication. In Sledge, GW and Roth BJ. Cisplatin in the management of breast cancer. *Semin. Oncol.*, **16**, 110-115, 1989.

O'BRIEN MER, TALBOT DC AND SMITH IE. (1993). Carboplatin in the treatment of advanced breast cancer: a phase II study using a pharmacokinetically guided dose schedule. *J. Clin. Oncol.*, **11**, 2112-2117.

POWLES TJ, JONES AL, JUDSON IR, HARDY JR AND ASHLEY SE. (1991). A randomised trial comparing combination chemotherapy using mitomycin C, mitozantrone and methotrexate (3M) with vincristine, anthracycline and cyclophosphamide (VAC) in advanced breast cancer. *Br. J. Cancer*, **64**, 406-410.

SLEDGE GW Jr, LOEHRER PJ Sr, ROTH BJ AND EINHORN LH. (1988). Cisplatin as first-line therapy for metastatic breast cancer. *J. Clin. Oncol.*, **6**, 1811-1814.

SMITH IE, WALSH G, JONES AL, PRENDIVILLE J, JOHNSTON S, GUSTERSON B, RAMAGE F, ROBERTSHAW H, SACKS N, EBBS S, MCKINNA JA AND BAUM M. (1995). High complete remission rates with primary neoadjuvant infusional chemotherapy for large early breast cancer. *J. Clin. Oncol.*, **13**, 424-429.

TORMEY DC, GELMAN R, BAND PR, SEARS M, ROSENTHAL SN, DEWYS W, PERLIA C AND RICE MA. (1982). Comparison of induction chemotherapies for metastatic breast cancer. *Cancer*, **50**, 1235-1244.

WORLD HEALTH ORGANIZATION (WHO). (1979). *WHO Handbook for Reporting Results of Cancer Treatment*, WHO offset publication No. 48. WHO: Geneva.